# A Decade of Evidence on Broiler Chicken Dead-on-Arrival Rates and Risk Factors: A Scoping Review

**DOI:** 10.3390/ani16050805

**Published:** 2026-03-05

**Authors:** Samantha Vitek, Leonie Jacobs

**Affiliations:** School of Animal Sciences, Virginia Tech, 175 West Campus Drive, Blacksburg, VA 24061, USA; samanthav26@vt.edu

**Keywords:** animal welfare, broiler chicken, dead-on-arrival, transport

## Abstract

The preslaughter phase for broiler chickens is distressing and can result in death prior to slaughter. The severity of this animal welfare concern warrants the exploration of the rates and risk factors. The aim of this scoping review was to synthesize current knowledge on rates and associated farm, flock, and preslaughter risk factors for dead-on-arrivals (DOA). Peer-reviewed experimental or observational studies were included that were written in English, published between January 2014 and December 2024, and that reported broiler chicken DOA with rates or associated risk factors in Google Scholar and ScienceDirect. A total of 344 articles were identified, and 24 articles met the eligibility criteria. Mean DOA rates ranged from 0 to 0.85%. In total, nine on-farm or flock-level and 11 preslaughter risk factors were identified, which could be categorized under four major causes of DOA: poor health, distress, thermal stress, and trauma. The risk factors most commonly identified were journey duration and distance, season, ambient temperature, lairage duration, and body weight. The findings highlight multiple opportunities to reduce DOA, including greater consideration of flock characteristics in preslaughter decision making, growing flocks that are at reduced risk of DOA, improvements in catching and loading practices, and better alignment of preslaughter management with environmental conditions.

## Simple Summary

Broiler chickens can experience significant distress during the period before slaughter, and some birds die before they arrive at the slaughter plant. These early deaths raise serious animal welfare concerns. The aim of this review was to examine existing research to understand how often these deaths occur and what factors increase the risk. We examined published studies from the past decade that reported rates and factors linked to these deaths. The review identified several risk factors that could be grouped into four main areas: poor bird health, distress, exposure to extreme temperatures, and physical injury. Factors related to flock health, catching and loading methods, transportation duration and distance, and environmental conditions were especially important. Overall, this review shows that many factors can contribute to an early death, but there are also opportunities to reduce the issue. Consideration of the bird’s health, careful handling, reducing duration, and adjusting practices to weather conditions can improve animal welfare and reduce death rates.

## 1. Introduction

When broiler chickens reach the desired body weight, they are caught and placed in crates, loaded onto a truck, transported to the slaughter plant, may be placed in lairage, unloaded, and finally slaughtered [1,2,3]. This multi-stage process, excluding slaughter itself, can be referred to as the preslaughter phase and exposes broilers to many animal welfare risks [4].

The catching process is mostly performed manually, but some companies apply mechanical catching methods. Manual catching involves the inverted carrying of broiler chickens by either one leg, with three to five broilers in one hand and one or two broilers in the other [5], or by both legs, with a maximum of two broilers per hand [6]. Alternatively, and rarely, broilers are manually caught, kept upright with a grip over the wings, carrying two birds at a time. When birds are mechanically caught, a vehicle with a conveyor belt or rotating ‘rubber fingers’ is driven through the house to catch and place birds in crates [7]. These birds are never inverted and are not manually handled or carried [7].

Birds are typically crated in plastic ‘drawers’ within metal containers that are loaded into the vehicle [8]. Once birds are crated and loaded onto the truck, they are driven to the slaughter plant. These vehicles may contain tarps, curtains or ventilation doors to protect birds from extreme weather, but in some cases and countries these are not utilized [9].

Upon arrival at the slaughter plant, the crated birds may remain on the truck or in a holding facility prior to processing, which is lairage. Lairage aims for birds to recover from transportation stressors and ensures that there is a supply of broilers for the slaughter process to continue without interruption [9,10]. The lairage duration may vary depending on plant-specific operations [9].

In slaughter plants with atmospheric stunning, containers or drawers enter the gas stunning chamber where broilers are rendered insensible before being removed and shackled [11]. In slaughter plants with electrical stunning, containers are often tilted and birds removed from crates while sensible, then shackled and stunned in a water bath prior to slaughter [11]. Slaughter then involves a neck cut for exsanguination aimed to induce rapid and complete death [11].

The preslaughter phase can result in death before slaughter, referred to as dead-on-arrival (DOA). DOA are broilers found dead at the time of unloading or shackling at the processing plant. These mortalities reflect a potentially severe animal welfare issue and are caused by poor health [12], distress [13], thermal stress [14], or physical trauma [12]. Poor flock health can predispose birds to DOA, with infectious diseases recorded in about 65% of DOA broilers, including laryngitis, tracheitis, fibrinous polyserositis, and purulent arthritis [15]. Distress during the preslaughter phase can induce cardiac arrhythmia, resulting in sudden death syndrome (thus, DOA) [13]. Nearly 60% of DOA broilers were found with lung congestion, attributed to this sudden death syndrome [13]. The thermal load imposed upon broilers during loading, transportation and lairage results in DOA due to either heat [16,17,18] or cold stress [17,19,20], accounting for up to 40% of deaths [14]. Nearly 10–30% of DOA broilers died due to internal injury caused by trauma, including liver ruptures, fractures, muscular trauma, and head trauma [13,15,21].

Aside from the animal welfare concern, DOAs have direct economic consequences [4,17]. These birds are condemned as unfit for human consumption, representing a direct loss of income, although they can be used for non-human consumption in certain conditions. A typical industry goal is to keep the DOA below 0.20% [14], which is an estimated annual loss of around 1.87 million broilers when considering the annual United States (U.S.) production of 9.33 billion broilers [22]. Globally, DOAs represent an estimated economic loss between $117 and $450 million annually [23], which is dependent upon the market and countries. In the U.S., a 0.20% DOA rate, with a complete loss of income, is estimated at $1.8 million loss annually [23].

The severity of this animal welfare concern and the direct economic consequences warrant a better understanding of risk factors associated with DOA. The objective of this scoping review was to synthesize current knowledge on rates and associated flock, farm, and preslaughter risk factors for DOA based on research published in the last decade.

## 2. Materials and Methods

The methodology for this scoping review followed the PRISMA-ScR framework to ensure transparent and reproducible reporting of the search strategy, study selection, and data synthesis (see Supplemental Material for PRISMA-ScR checklist) [24]. No review protocol was developed or registered for this scoping review. The scoping review included English-language articles on broiler chicken dead-on-arrival rates during the preslaughter phase and associated risk factors, published between 1 January 2014 and 31 December 2024. For this scoping review, the term “article” refers to a publication identified via the search process. The search for possible eligible articles was conducted using Google Scholar and Science Direct databases using the term “broiler chicken” AND “dead-on-arrival”. The last search using these keywords was 25 January 2025. The identified articles from each database were exported to Microsoft Excel to document the title. Duplicate articles originating from the search were removed manually.

Consistent with PRISMA-ScR guidance, the screening process occurred in two stages: (1) title and abstract screening and (2) full-text review. Reasons for exclusion were documented at both stages. The titles and abstracts were reviewed to identify the articles that contained information on DOA rate or risk factors. This was followed by reviewing the full article to decide on inclusion or exclusion. The authors, year, journal, and database were included in the Microsoft Excel workbook for eligible articles. Eligibility criteria for inclusion for the final selection were as follows:

1. Only broiler chickens as animal species. Articles on broiler breeders were excluded from the review.
2. Articles that reported studies on DOA, with associated rate and/or risk factor(s) during the preslaughter phase, including catching, transport, lairage, or articles collecting data from early life through slaughter.
3. Articles that presented original research, which were peer-reviewed and published online.

Exclusion criteria for the final selection were as follows:

1. An animal species other than broiler chickens.
2. Articles that reported on DOA without their rate or associated risk factor(s).
3. Articles that were not peer-reviewed, were not original research articles, and not published online as a full journal article (such as graduate student thesis or dissertations, conference proceedings, book or book chapters, literature reviews, or reports).

The study design was not used as an exclusion criterion; experimental and epidemiological articles were considered equally during selection. During data extraction, reported risk factors and their statistical associations with DOA were recorded. For this review, only statistically significant associations (*p* < 0.05), as defined in the original article, are described in the synthesis of risk factors. When a study evaluated a risk factor but did not find a statistically significant association with DOA, this is noted in Section 3. The data were independently extracted by one reviewer and double-checked to ensure accuracy. As the objective of this scoping review was to synthesize current evidence, no critical appraisal or risk of bias assessment was performed.

## 3. Results

**Figure 1:**
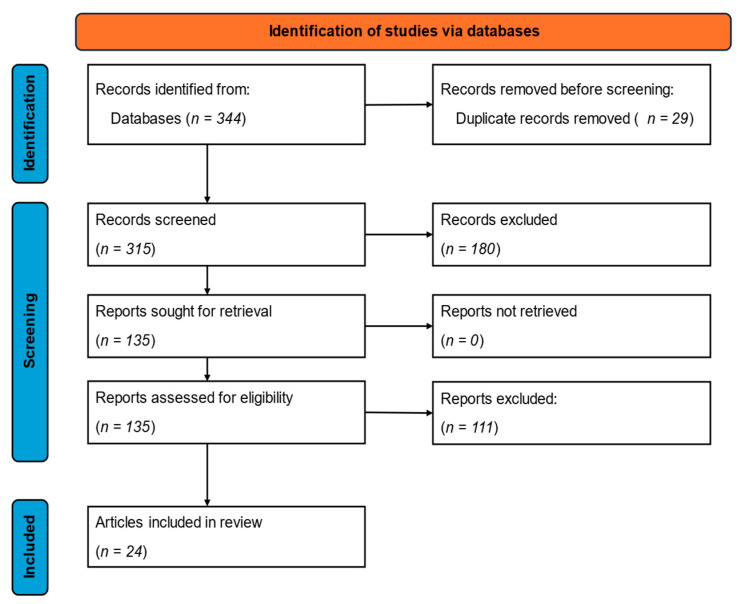
Flow diagram for this scoping review on rate and risk factors for dead-on-arrivals in broiler chickens.

We recognize that not all relevant publications may have been included with a bias toward English-language publications and peer-reviewed research articles. A total of 344 articles were identified (Figure 1) relating to DOA. Of these, 29 were duplicates. The remaining 315 articles were screened for eligibility. Out of 315 articles, 291 articles were excluded with 24 articles remaining for inclusion in the scoping review (10 from Science Direct and 14 from Google Scholar).

The reported DOA rate means ranged between 0% and 0.85%, with a minimum reported rate of 0% and a maximum reported rate of 27.56% (Table 1). Nineteen flock, farm, and preslaughter risk factors were identified for DOA and are presented in Table 2.

### 3.1. Risk Factors Related to On-Farm Conditions or Flock Characteristics

Nine on-farm or flock risk factors for DOA were identified, including the parental flock age, genetic strain, bird sex, bird age, body weight, on-farm mortality, stocking density, flock size, and drinking system (Table 2).

#### 3.1.1. Parental Flock Age

The parental flock age (breeder flock age) was assessed in one out of 24 articles and was identified as a risk factor. In an epidemiological study consisting of 25,476 loads, broilers from parent flocks of mixed ages had a 13% higher DOA rate compared to those from parent flocks of peak age [16].

#### 3.1.2. Genetic Strain

Genetic strain was identified as a risk factor in three articles [16,26,29]. The DOA rate was higher for Ross 308 birds (0.063%) compared to Hubbard JA787 birds (0.015%) in an epidemiological study in Norway [26]. In line, Ross 308 DOA rates were 73% higher compared to Hubbard JAs and 6% higher than Cobbs in the United Kingdom [16]. In The Netherlands, conventional broilers had higher DOA rates compared to two strains of slow-growing broilers [29].

#### 3.1.3. Sex

Sex was associated with DOA in two articles, with males more at risk than mixed flocks or females. At 38–39 days of age, males and mixed-sex loads had a higher risk of DOA compared to females in a study conducted in Canada [17]. In Thailand, higher DOA rates were reported in male broilers compared to mixed-sex flocks, while females showed a lower DOA incidence; this article included broilers slaughtered between 40 and 71 days of age [34].

#### 3.1.4. Age

The relationship between bird age and DOA varied in the four articles that identified it as a risk factor [16,17,19,34]. Higher DOA rates were observed in older broilers (56–71 days) in Thailand [34], in broilers > 38 days old in the UK [16]. In contrast, young broilers (≤39 days) had higher DOA rates in Turkey [19] and in Canada [17], while no association between age and DOA was found two Norwegian studies [26,42].

#### 3.1.5. Body Weight

Increased body weight was associated with higher DOA rates [17,30,33,35,41], with birds > 2.28 kg having a higher risk than those ≤2.14 kg. However, no association was found in a Norwegian study [26].

#### 3.1.6. On-Farm Mortality

High on-farm mortality rates were associated with increased DOA rates in Canada, UK, and Thailand [16,30,34]. However, no association was found in studies in Norway and Canada [17,26,42]. In contrast, a Belgian study reported that DOA rates decreased with increasing mortality rates [37].

#### 3.1.7. Stocking Density

Stocking density during rearing was identified as a risk factor for DOA in one article. For each 1 kg/m^2^ decrease in stocking density, the DOA rate increased by 0.026% [32]. In Canada, this was not identified as a risk factor [17].

#### 3.1.8. Flock Size

One article identified small flocks at higher risk of high DOA rates compared to large flocks [42].

#### 3.1.9. Drinker Type

In one article, flocks housed with cup or bell drinkers had a higher risk of DOA compared to flocks with nipple drinkers [35].

### 3.2. Risk Factors Related to Preslaughter Conditions

Eleven preslaughter risk factors were identified for DOA, including the feed withdrawal time, catching method, catching team, light adaptation during catching, crate stocking density, time of day for catching, journey duration and distance, condition of the birds upon arrival at lairage, lairage duration, ambient (external) temperature, and season.

#### 3.2.1. Feed Withdrawal

Four articles out of 24 identified longer feed withdrawal times as a risk factor for increased DOA rates, as reported in two Thai studies [32,34] and two Canadian studies [17,30]. The DOA rate increased with the increase in feed withdrawal time [32,34]. In line, the DOA rate increased when feed withdrawal was longer than 6 h [30]. However, a quadratic effect of the feed withdrawal time on DOA was reported in one study, with the risk lowest after 8 h of feed withdrawal before loading, but higher with 0 h of feed withdrawal or with 19 h of feed withdrawal [17].

#### 3.2.2. Catching Method

The mechanical catching of broilers resulted in increased DOA rates in two studies [16,29]. Broilers caught using mechanical catching had a 32% increase in DOA rates compared to those caught manually [16]. Mechanically caught broilers had a mean DOA rate of 0.15%, while manually caught broilers had a mean DOA rate of 0.10% [29].

#### 3.2.3. Catching Team

The choice of catching team mattered, with the team identified as a risk factor for DOA in one article. The risk of DOA differed between two catching teams but not the other six catching teams [17].

#### 3.2.4. Light Adaptation During Catching

The use of red lights during catching increased the risk of DOA compared to decreased light intensity in one study [35]. The use of blue lights had no effect [35].

#### 3.2.5. Crate Stocking Density

The crate stocking density influenced the risk of DOA in three studies. At <−14 °C, densities below 40 kg/m^2^ increased the DOA risk compared to 40–45 kg/m^2^, with the risk rising by 0.01% per °C decrease [17]. Above −4 °C, the crate stocking density had no effect [17]. No association between the crate stocking density and DOA was found in Germany or Pakistan [28,39], while increasing crate density was associated with increased DOA rates in Thailand [32,34].

#### 3.2.6. Time of Day for Catching

Three out of 24 articles identified time of day as a risk factor. Higher DOA rates were observed during daytime transports compared to morning and night [32,34], and midday transports showed the highest rate relative to morning, evening, and night [28].

#### 3.2.7. Journey Duration and Distance

Longer [34,36,42] and further transports [10,16,31,33] increased the risk for DOA in most but not all studies that included this factor. This duration effect was noted only after midnight in one article [2]. In Canada, the risk of DOA rose by 0.045% per hour of transport [17], and journeys exceeding 60 min had 6% higher DOA rates compared to shorter trips [16]. In line, transport distance was positively associated with DOA in Spain, particularly for mixed-sex loads in winter [36]. Conversely, two articles either reported no relationship [39] or observed a somewhat opposite relationship. In the Czech Republic, distances longer than 100 km and shorter than 50 km had an increased risk of DOA compared to intermediate distances [20].

#### 3.2.8. Condition of Birds on Arrival at Lairage

Higher DOA rates were observed in loads containing wet birds compared to those with dry birds [17].

#### 3.2.9. Lairage Duration

Longer lairage can increase DOA rates based on studies in Thailand [34], Canada [17], Turkey [19], Spain [33], and in the UK [16]. This effect was also time-dependent, with increased DOA rates seen only in broilers caught after midnight [2]. However, the lairage duration was assessed but not identified as a risk factor in one Turkish study [41].

#### 3.2.10. Ambient (External) Temperature

The ambient (external) temperature can increase DOA rates if it is too low or too high, according to five articles [16,17,19,20,26]. In the UK, the DOA risk was 16.9 times higher with ambient temperatures above 30 °C and progressively lower at 25–30 °C (3.9 times) and 20–25 °C (1.2 times), compared to 10–15 °C [16]. In contrast, in Canada, DOA decreased by 0.03% for each 1 °C increase in average ambient temperature [17]. In Norway, Turkey, and the Czech Republic, the highest DOA rate occurred at low temperatures [19,20,26]. However, in Germany, no relationship was observed with temperatures ranging from −6.6 °C to 28.1 °C [28].

#### 3.2.11. Season

With ambient temperatures impacting the DOA rates, it is unsurprising that season was associated with DOA in nine out of 24 articles, although the results are not fully aligned. Higher DOA rates were reported in fall compared to spring [17], in winter compared to summer [30], in fall and winter compared to spring and summer [28], in spring and summer compared to fall and winter [29], and in winter compared to other seasons [10,20]. The DOA rates were higher in fall compared to summer, spring, and winter in Spain [36], and in Thailand, DOAs were higher in winter and lower in summer compared to the rainy season [32,34].

## 4. Discussion

This scoping review gathered up-to-date knowledge on DOA rates and associated risk factors, focusing on peer-reviewed studies published between 2014 and 2024. The mean DOA rates were relatively low (0–0.85%) but ranged to an extreme value of 27.6% in one transport. This was an extreme case for one transport that was not addressed in the original article [41]. Nine on-farm or flock characteristics (parental flock age, genetic strain, bird sex, bird age, body weight, on-farm mortality, stocking density, flock size, and drinker system) and 11 preslaughter risk factors (feed withdrawal time, catching method, catching team, light adaptation during catching, crate stocking density, time of day for catching, journey duration and distance, condition of birds on arrival at lairage, lairage duration, ambient temperature, season) were identified for this welfare concern, highlighting its multifactorial nature. Most articles (10) identify journey duration and distance as a risk factor for DOA, followed by season (9), ambient temperature (5), lairage duration (5), and body weight (5), feed withdrawal time (4), on-farm mortality (4), age (4), genetic strain (3), crate stocking density (3), time of day for catching (3), catching method (2), sex (2), parental flock age (1), drinker type (1), on-farm stocking density (1), flock size (1), catching team (1), and light adaptation during catching (1).

Four major causes of death were identified through necropsies previously, namely poor health, distress, thermal stress, and trauma [12,13,14,15,16,17,18,19,20,21], and these strongly align with the risk factors for DOA identified in research in the past ten years. The risk factors identified in the current review can be categorized under those groups directly or indirectly. Understanding how these factors interact within and across categories is essential for identifying key interventions to reduce DOA rates.

### 4.1. Risk Factors Related to On-Farm Conditions or Flock Characteristics

#### 4.1.1. Poor Health

The health and resilience of broiler chickens can predispose them to die during the preslaughter phase, and these factors are influenced by on-farm and flock conditions. Broilers at higher risk of dying during the preslaughter phase include those from parent flocks that are not at peak age (32–50 weeks of age), those from fast-growing strains, males, young or old birds, and heavier broilers. The parent flock age can impact the chick quality and thus the health throughout the broiler’s life [43]. Fast-growing broilers are more at risk than slow-growing broilers because the latter are robust, better able to handle heat stress, and have reduced risk for health issues [29]. Males typically have higher body weights and are more susceptible to thermal stress [14] and sudden death syndrome [44], which may explain the higher DOA rate compared to females. As broilers age and gain weight, their ability to thermoregulate decreases, making them more susceptible to heat stress especially [45]. In addition, their relatively small heart and lung sizes [46] cause difficulty maintaining circulation, and under distress this can lead to heart failure and fluid accumulation in the lungs and abdomen [13], respiratory distress, and subsequent death [47]. Birds’ quality and health on farm appear to predict their resilience to stressors during the preslaughter phase, supported by high on-farm mortality (especially natural deaths rather than culls) serving as a rough indicator of flock health [48], although one study reported the opposite [37]. This discrepancy may reflect differences among countries in laws, infrastructure, transport conditions, and attitudes toward animal handling and welfare. Most evidence from these studies suggests that birds’ health status and housing conditions can inform management decisions for the preslaughter phase, and modifying these factors may theoretically result in the reduction in DOA rates. The use of slow-growing broilers could reduce preslaughter losses, as they are more tolerant to heat stress and less prone to hereditary health issues [29]. Housing only females may also be beneficial, as they generally have fewer health issues and lower susceptibility to thermal stress compared to males [14,44]; however, this may not be feasible. Additionally, reducing the slaughter age and target weight could benefit broiler health, since many health concerns are age- or weight-dependent. The flocks that are at higher risk of DOA may benefit from adjustment during catching and transport, including more careful handling, reducing stocking density in crates, improved microclimate conditions, scheduling transports during the cooler parts of the day, and limiting journey duration where possible. High on-farm mortality rates may indicate underlying health or management problems [48] and could be used as a practical indicator for implementing adjustments during catching and transportation to reduce DOA risk.

#### 4.1.2. Housing

Housing conditions were associated with DOA, which may be due to their impact on a flock’s health status, although the evidence is limited or inconsistent. A low stocking density, which generally leads to increased feed intake and heavier birds [49,50], increased the risk of DOA. Older studies (not included in the review) reported no association between on-farm stocking density and DOA [17,51,52]. Small flocks had higher DOA rates [42]; however, the numerical difference in flock size between the “normal DOA” category (mean DOA < 0.30%; mean flock size 18,621) and the “high DOA” category (mean DOA > 0.30%; mean flock size 17,858) was small (763 birds) [42]. Older studies that were not included in the review reported an opposite association, with larger flocks at higher risk of DOA [1,51]. Considering this limited and conflicting evidence, it is unlikely that increasing the flock size would reduce the DOA rates.

Access to cup or bell drinkers increased the risk of DOA, although only 10% of flocks in this study had those drinker types [35], and no other studies included drinker type in their risk analysis. However, cup or bell drinkers have a higher risk of bacterial contamination compared to nipple drinkers [53], which can then lead to infection and possibly death. Since the evidence here is limited, changing the drinker type may not be an effective strategy to reduce the DOA risk.

### 4.2. Risk Factors Related to Preslaughter Conditions

#### 4.2.1. Distress

Broilers are more likely to die during the preslaughter phase when subjected to long feed withdrawal, mechanical catching, red light during catching, and when certain catching teams are employed. In addition, longer further transports and long lairage exacerbate this risk of DOA by prolonging exposure to stressors.

Prolonged feed and water withdrawal can lead to a negative energy balance and dehydration, reducing the birds’ ability to cope with temperature and transportation stressors [54,55], explaining the association between these factors and DOA. Broilers that are unfit for transport or already dead are typically removed by the catching team during manual catching [56]. However, mechanical catching can lack this selection mechanism, unless the catching crew walks through the house to do this beforehand. This means that unfit or dead birds may be transported to the slaughter plant and later recorded as DOA, artificially increasing the DOA rate, even if the preslaughter phase itself did not increase mortality [29,56]. Variation in catching team training and company protocols might further influence bird handling and contribute to DOA risk [17], potentially through increased fear and distress during handling [57,58]. Longer further journeys and longer lairage duration prolong birds’ exposure to stressors, such as feed and water withdrawal, adverse environmental conditions (heat, cold, rain, wind), vibration and vehicle motion [59], and crowding [9]. Extended exposure to these stressors increases the likelihood of birds dying during the preslaughter phase.

There are opportunities to improve animal welfare in the preslaughter phase. Evidence suggests that feed and water withdrawal timing should be carefully managed to prevent prolonged fasting and dehydration [54,55]. For mechanical catching, best practice involves the removal of unfit and dead birds beforehand to improve bird welfare and avoid inflated DOA rates [29,56]. This review also identified opportunities for catching crew training to avoid stress-induced mortality. Coordinating slaughter timing with processing logistics may help reduce transportation distances and lairage durations, thereby lowering the risk of DOA. Additionally, reconsidering lairage conditions to ensure protection from extreme weather can further minimize exposure to stressors, although only one study indicated that wet birds at lairage were at greater risk. Together, these measures highlight the importance of integrated management across all preslaughter stages to reduce the DOA rates and improve animal welfare.

#### 4.2.2. Thermal Stress

Both under- and overstocking crates, transport during midday heat, long journeys, and long lairage may compromise bird survival during the preslaughter phase, especially when birds are transported outside of their thermoneutral zone. Low crate stocking density during extreme cold temperatures poses a risk for hypothermia [17]. Under these cold conditions, overstocking might be beneficial, since the metabolic heat produced by the birds would allow for buffering against cold weather [17,60]. In contrast, high stocking density can be detrimental at high temperatures, predisposing birds to death from hyperthermia [17,34,60]. High stocking densities can also lead to birds piling on top of each other and suffocating [9]. Heat stress may therefore pose a higher risk for DOA, which is more likely during midday transports, as elevated body temperatures and ineffective panting lead to physiological disturbances such as elevated plasma creatine kinase activity, respiratory alkalosis, and acid–base regulation that can result in death [61,62]. Again, longer journeys and lairage exacerbate these thermal stressors, further increasing the mortality risk [17]. Although active ventilation is typically absent during transport, some thermal stressors during lairage can be mitigated through ventilation management.

A large body of evidence links broiler welfare and DOA to thermal stressors during the preslaughter phase. The effective management of the crate stocking density, journey scheduling, and ventilation during lairage is critical to minimizing thermal stress and improving preslaughter welfare outcomes.

#### 4.2.3. Physical Trauma

In addition to causing distress, some conditions risk physical trauma. Broiler chickens subjected to mechanical catching, red lighting, and daytime handling were at increased risk of preslaughter mortality, with DOA rates also dependent on the catching teams. We suggest that these increased risks are, in part, due to a higher likelihood of physical trauma under those conditions. Mechanical catching was associated with an increased rate of injuries [7,56]. The rate of injuries can vary depending on the model of harvester or the fitness of the flock, with unfit birds more susceptible to injuries [7,56]. If birds are more active, like under red light compared to blue lights [63], or under bright (daylight) light compared to dim lighting [64,65,66], catchers will likely have more difficulty collecting birds, increasing the risk of injuries and DOA [35]. However, in the current review, only one paper supported the impact of lighting, limiting our ability to draw conclusions related to this aspect. The evidence does support the importance of appropriate training of catching teams, since this will directly impact how broilers are handled and loaded into transport crates, which will directly impact injuries [17]. However, training efficacy studies are lacking. If trauma occurs from catching and loading, prolonged journeys and lairage increase the risk of birds succumbing to their injuries rather than the journey itself directly causing trauma [17].

These findings highlight that factors associated with physical trauma contribute significantly to increased mortality during the preslaughter phase. Reducing handling distress and minimizing injury are therefore key strategies for lowering DOA rates. Careful selection and training of catching teams, optimizing mechanical harvesting systems, catching under lighting conditions that reduce bird activity, and minimizing journey duration and lairage time can all be effective approaches to achieve this. Implementing these measures may substantially improve broiler chicken welfare and reduce preslaughter losses.

### 4.3. Study Limitations and Avenues for Future Research

Although nine flock characteristics and 11 preslaughter factors were identified as influencing the DOA rate, the findings across studies showed inconsistencies in associations between risk factors and DOA, including age, body weight, and on-farm mortality for flock characteristics and feed withdrawal, crate stocking density, journey duration, distance, ambient temperature, and season for preslaughter characteristics.

Since the impact of the crate stocking density on DOA is temperature-dependent, the association may be obscured under moderate temperatures or when stocking density is systematically lower in hot conditions and higher in cold conditions. This may explain why some studies did not observe this association, for instance [39], where temperatures ranged between 3.6 and 9.5 °C or for temperatures above −4 °C [17].

Slaughter practices will differ, resulting in varying relationships between risks and DOA. For example in Norway, where broilers are typically slaughtered much younger (27–37 days) than some other countries (up to 71 days in Thailand) [16,19,34], no association between age, weight and DOA was found [26,42]. This could reflect the higher resilience to stressors when birds are younger [45].

There is limited evidence for the effects of parental flock age, drinker type, on-farm stocking density, catching team, light color during catching, and (wet) condition of the birds at lairage on DOA, with only one study identifying these as risk factors. No other selected studies in this scoping review included these variables in their analysis (except for on-farm stocking density), highlighting the need to include them in future studies to confirm potential associations.

Future research should further explore the potential interaction effects between risk factors. Interactions such as age × sex, crate stocking density × ambient temperature, and lairage duration × time of day may better reflect the multifactorial nature of DOA risk. In addition, more detailed monitoring of transport microclimate conditions within crates (e.g., temperature, humidity, and airflow) could improve understanding of their conditions during transport. Finally, predictive approaches, including machine learning models, may offer opportunities to improve DOA risk assessment and support proactive management strategies.

This review included publications between 2014 and 2024, which may have resulted in the omission of some impactful studies that were published outside this period. Limiting the search to English-language publications may have also introduced a language bias, potentially excluding relevant findings from non-English sources. Together, this may have introduced a selection bias, which may impact the strength or direction of the review’s conclusions.

## 5. Conclusions

This scoping review identified 20 risk factors associated with broiler chicken DOA. Across the articles included, the mean DOA rates were <1%, primarily driven by distress and thermal stress, and associated most frequently with journey duration and distance. Based on these findings, several management implications can be drawn for the broiler chicken industry:

- Flock characteristics should be considered as part of slaughter planning and decision-making to reduce DOA rates, or flock characteristics can be chosen to limit the DOA risk. The high-risk group (broilers from fast-growing strains, males, young or old birds, heavy birds and flocks with high on-farm mortality rates) may need different strategies than the low-risk group (birds from slow-growing strains, females, intermediate-aged birds, light birds, and flocks with low on-farm mortality rates).
- Considerations during catching and loading include limiting the feed and water withdrawal times to prevent prolonged fasting and dehydration, refinement of mechanical catching including removing unfit or dead birds prior to catching, training catching teams, catching during the early morning or nighttime to reduce bird activity, adjusting the crate stocking densities according to the ambient temperature, and limiting transportation and lairage durations to subsequently reduce the DOA rates.
- Adjusting preslaughter management to environmental conditions, by scheduling transportation during cooler parts of the day in warmer seasons, shortening the transportation and lairage times, improving the lairage conditions and preparing for seasonal or temperature extremes are all important.

Together, these management interventions may significantly reduce the DOA rates and improve animal welfare outcomes.

## Figures and Tables

**Table 1:** Overview of studies included in the review, with mean dead-on-arrival rates (DOA) and range (if provided) in ascending order, with study design (experimental (EX) or epidemiological (EP)), sample size (*n*), and country.

Study	Study Design	Sample Size (*n*)	Country	DOA % [Range]
Sans et al., 2014 [25]	EX	10 farms	Brazil	0.0
Forseth et al., 2024 [26]	EP	4228 flocks	Norway	0.063 [0.024–0.082] (Ross 308) 0.015 [0.0–0.02] (Hubbard JA787)
Kittelsen et al., 2017 [27]	EP	59 flocks	Norway	0.07 [0.01–0.36]
Allen et al., 2023 [16]	EP	25,476 loads	United Kingdom	0.08 [0.0–17.39]
Gickel et al., 2024 [28]	EP	14,054 loads	Germany	0.09 [0.0–3.36]
Van Emous et al., 2024 [29]	EP	21,540 flocks	Netherlands	0.14 (fast-growing) 0.09 (slow 1) 0.55 (slow 2)
Cockram et al., 2019 [30]	EP	212 loads	Canada	0.13
dos Santos et al., 2020 [31]	EX	12 loads	Brazil	0.17
Pirompud et al., 2022 [32]	EP	211 flocks	Thailand	0.18 [0.04–0.46]
Villarroel et al., 2018 [33]	EP	1856 flocks	Spain	0.18 [0–0.99]
Pirompud et al., 2023 [34]	EP	13,581 loads	Thailand	0.20 [0.03–1.02]
Van Limbergen et al., 2020 [35]	EP	2309 flocks	7 EU countries	0.20 [0.01–4.60]
Averos et al., 2020 [36]	EP	2103 loads	Spain	0.26
Saraiva et al., 2020 [2]	EP	64 loads	Portugal	0.29 [0.02–1.89]
Jacobs et al., 2017a [37]	EP	81 flocks	Belgium	0.30 [0.04–3.34]
Saraiva et al., 2024 [38]	EP	70 loads	Portugal	0.30 [0.0–2.83]
Hussnain et al., 2020 [39]	EX	9 flocks	Pakistan	0.33
Teke, 2019 [19]	EP	4062 loads	Turkey	0.389
Valkova et al., 2022 [40]	EP	1,094,054,474 broilers	Czech Republic	0.425
Vecerek et al., 2016 [20]	EP	247,925,689 broilers	Czech Republic	0.47 [0.31–0.72]
Caffrey et al., 2017 [17]	EP	2007 loads	Canada	0.50 [0.0–19.4]
Tekindal et al., 2023 [41]	EP	26,599 loads	Turkey	0.57 [0.0–27.56]
Kittelsen et al., 2017 [42]	EP	61 flocks	Norway	0.85 [0.01–2.26]
Teke et al., 2019 [10]	EX	12 flocks	Turkey	Not reported

**Table 2:** Overview of identified flock, farm, and preslaughter risk factors for dead-on-arrival (DOA) broilers, including the number of studies identifying significant associations (*p* < 0.05), the number of studies that report no association, and the direction of effect for an increased risk for DOA (↑ indicates a positive association; ↓ indicates a negative association).

Risk Factor	Studies Reporting an Association (*n*)	Studies Reporting No Association (*n* and [Reference])	Condition with Increased Risk for DOA
Journey duration and distance	10	1 [39]	↑ duration [17,34,36,42] ↑ distance [2,10,16,20,31,33] ↓ distance [20]
Season	9	0	Winter vs. summer [30]; vs. any [10,20]; vs. rainy season [32,34] Fall vs. spring [17]; vs. any [36] Fall and winter vs. spring and summer [28] Spring and summer vs. fall and winter [29]
Ambient temperature	5	1 [28]	↑ [16] ↓ [16,17,19,20,26]
Lairage duration	5	1 [41]	↑ [16,17,19,33,34]
Body weight	5	1 [26]	↑ [17,30,33,35,41]
Feed withdrawal time	4	0	↑ [17,30,32,34]
On-farm mortality	4	3 [17,26,42]	↑ [16,30,34] ↓ [37]
Flock age	4	2 [26,42]	↑ [16,34] ↓ [17,19]
Genetic strain	3	0	Ross 308 vs. Hubbard JA787 [26] Ross 308 vs. Hubbard JA and Cobb [16] Conventional vs. slow-growing [29]
Crate stocking density	3	2 [28,39]	↑ [32,34] ↓ [17]
Time of day for catching	3	0	Daytime vs. morning and night [32,34] Midday vs. morning, evening, and night [28]
Catching method	2	0	Mechanical vs. manual [16,29]
Sex	2	0	Male and mixed-sex vs. females [17] Males vs. mixed-sex [34]
Parental flock age	1	0	Mixed ages vs. peak age [16]
Drinker type	1	0	Cup or bell drinkers vs. nipple drinkers [35]
Stocking density	1	1 [17]	↓ [32]
Flock size	1	0	Small vs. large flocks [42]
Catching team	1	0	Difference between two teams [17]
Lighting during catching	1	0	Red lights vs. low light intensity [35]

## Data Availability

Data underlying this manuscript are made accessible through the Virginia Tech Data Repository at https://doi.org/10.7294/31438195.

## Supplementary Materials

The following supporting information can be downloaded at https://www.mdpi.com/article/10.3390/ani16050805/s1, Table S1: PRISMA-ScR Checklist.
